# Surface-Based Analysis on Shape and Fractional Anisotropy of White Matter Tracts in Alzheimer's Disease

**DOI:** 10.1371/journal.pone.0009811

**Published:** 2010-03-22

**Authors:** Anqi Qiu, Kenichi Oishi, Michael I. Miller, Constantine G. Lyketsos, Susumu Mori, Marilyn Albert

**Affiliations:** 1 Division of Bioengineering, National University of Singapore, Singapore, Singapore; 2 Clinical Imaging Research Centre, National University of Singapore, Singapore, Singapore; 3 Singapore Institute for Clinical Sciences, The Agency for Science, Technology and Research, Singapore, Singapore; 4 Department of Radiology, Johns Hopkins University School of Medicine, Baltimore, Maryland, United States of America; 5 Center for Imaging Science, Johns Hopkins University, Baltimore, Maryland, United States of America; 6 Department of Psychiatry, Johns Hopkins Bayview Medical Center and Johns Hopkins University School of Medicine, Baltimore, Maryland, United States of America; 7 Department of Neurology, Johns Hopkins University School of Medicine, Baltimore, Maryland, United States of America; Mental Health Research Institute of Victoria, Australia

## Abstract

**Background:**

White matter disruption has been suggested as one of anatomical features associated with Alzheimer's disease (AD). Diffusion tensor imaging (DTI), which has been widely used in AD studies, obtains new insights into the white matter structure.

**Methods:**

We introduced surface-based geometric models of the deep white matter tracts extracted from DTI, allowing the characterization of their shape variations relative to an atlas as well as fractional anisotropy (FA) variations on the atlas surface through large deformation diffeomorphic metric mapping (LDDMM). We applied it to assess local shapes and FA variations of twenty-three deep white matter tracts in 13 patients with AD and 19 healthy control subjects.

**Results:**

Our results showed regionally-specific shape abnormalities and FA reduction in the cingulum tract and the sagittal stratum tract in AD, suggesting that disruption in the white matter tracts near the temporal lobe may represent the secondary consequence of the medial temporal lobe pathology in AD. Moreover, the regionally-specific patterns of FA and shape of the white matter tracts were shown to be of sufficient sensitivity to robustly differentiate patients with AD from healthy comparison controls when compared with the mean FA and volumes within the regions of the white matter tracts. Finally, greater FA or deformation abnormalities of the white matter tracts were associated with lower MMSE scores.

**Conclusion:**

The regionally-specific shape and FA patterns could be potential imaging markers for differentiating AD from normal aging.

## Introduction

Diffusion tensor imaging (DTI) is a magnetic resonance imaging (MRI) technique that enables the measurement of the restricted diffusion of water in tissue. DTI has been widely applied in studies of Alzheimer's disease (AD) to obtain new insights into the tissue structure of brain white matter, including quantitative measurements of tissue properties such as diffusivity and fractional anisotropy (FA) derived from diffusion tensor [1], [2], [3], [4], [5], [6]. A number of studies have reported reduced FA and increased diffusivity in patients with AD in the fornix [5] and the cingulum bundle [1], [2], [3], [4], [5], [6]. Most of these studies have been limited to measurements of contrasts such as diffusivity and FA using manual region-of-interest (ROI) or voxel-based analysis. Evidence that shape analysis on gray matter structures (e.g. the hippocampus) distinguished patients with AD from healthy control subjects [7], [8], [9] suggests that geometric shapes of the white matter tracts may also give insights of the disease. Nevertheless, it is still challenging to study the geometry (such as shape) of the white matter tracts revealed by DTI and its relationship with AD because of difficulties in quantifying specific white matter structures visualized by the DTI acquisition. Therefore, this paper focused on surface models of shapes and FA of deep white matter tracts and identified their relationship with AD.

Adapting voxel-based morphometry used in structural MRI [10], the voxel-based analysis of DTI serves as an exploratory analysis to make statistical inferences about differences in diffusion properties of brain tissues in an atlas coordinate system. It first spatially normalizes the images of individual subjects (such as FA, T_1_ or T_2_) to an atlas' whole brain space where spatial smoothing and voxel-by-voxel statistical testing are then performed. Recent registration algorithms specialized for DTI have been developed and have shown to improve structural alignment when considering the tensor structure of DTI [11], [12], [13], [14], [15]. Voxel-based analysis in DTI has been widely used to identify FA and diffusivity abnormalities in a variety of clinical studies. Nevertheless, there is the need for spatial smoothing which makes localization of abnormalities challenging to interpret in terms of the white matter tracts. To address this issue, researchers [16], [17], [18], [19] characterized FA or diffusivity as functions indexed over manifolds such as curves, medial surfaces representative of the overall geometry of a white matter tract, and skeleton of the white matter. These demonstrated the potential for increased sensitivity in statistical analysis using geometrical models of the white matter tracts and tract-based analysis.

In this study, we followed the idea of the tract-based analysis and introduced the surface representation of twenty three deep white matter tracts that were defined based on Mori's white matter atlas [20]. We chose these deep white matter structures because they are reliably delineated from DTI and some of them are adjacent to gray matter structures (e.g., hippocampus) with anatomical abnormalities in AD. Perhaps, gray matter abnormalities could influence adjacent white matter structures during AD process. Using advanced brain mapping techniques, large deformation diffeomorphic metric mapping (LDDMM) [21], [22], the surface model of the white matter tracts was automatically constructed by transforming the shape of the atlas white matter tracts to individual subjects through a flow of diffeomorphisms. This surface model facilitated the study on local shape and FA variations of the white matter tracts. Given the important role of the hippocampal shape in distinguishing patients with AD from healthy control subjects, we expect that the white matter tracts connecting the hippocampus to the rest of the brain, such as the cingulum tract, would show regionally-specific shape abnormalities in AD. Additionally, we also expect regionally-specific pattern of FA reduction in AD as measurement of white matter tissue disruption. Compared with traditional ROI-based volumetric and mean FA analysis on the white matter tracts, the regionally-specific pattern of FA reduction and shape abnormalities would potentially increase statistical power for differentiating AD from healthy aging, which would be beneficial to clinical diagnosis.

## Results

**Figure 1:**
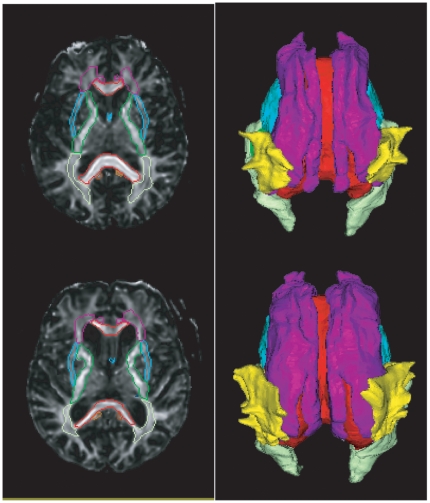
Examples of the white matter tract segmentation. Rows respectively illustrate the white matter tracts of a healthy elderly subject and a patient with AD. The left column shows the volume representation in the FA maps, while the right column shows the surface representation in the superior view.

**Figure 2:**
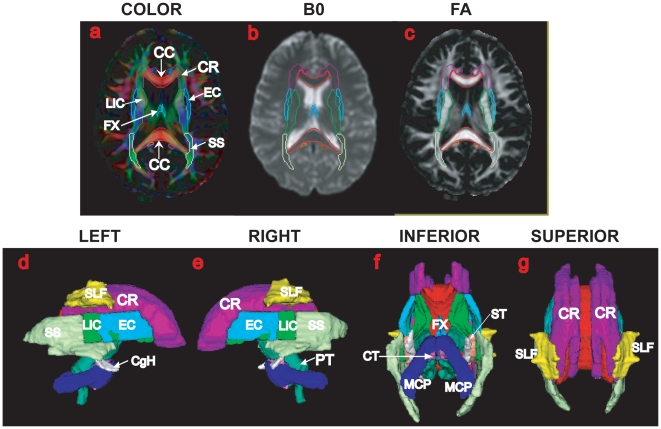
The single-subject atlas of the deep white matter tracts. The top row illustrates the color map, image without diffusion weighting, and fractional anisotropy (FA) with the contours of the deep white matter tracts, respectively. The bottom row shows the surface representation of the deep white matter tracts in the left, right, inferior, and superior views. Each tract surface is color coded. The anatomical definition of each white matter tract was detailed in [20]. The abbreviations of the tracts' names are given in Text S1.

We applied the surface-based analysis for assessing shape abnormalities of the deep white matter tracts in 19 normal comparison subjects and in 13 subjects with AD (Table 1). Shown in Figure 1 are examples of the white matter tracts extracted from the DT images of a healthy elderly (top row) and a patient with AD (bottom row) according to anatomical definitions given in Figure 2. Volume and surface representations are respectively shown in the left and right columns. Visually, the extracted deep white matter tracts clearly include the region with high FA. The enlarged lateral ventricles in the patient with AD did not influence the extraction accuracy of the commissural tract and other surrounding white matter tracts. The accuracy of this atlas-based diffeomorphic segmentation for extracting the white matter tracts has been validated using 237 manually labeled landmarks in the DT images of 13 AD patients and 18 healthy elderly subjects [21]. These DT images were a subset of the images used in this study. The landmarks were placed at the boundary of the white matter tracts [20]. In the healthy control subjects, 80% of deformed landmarks had distance to the manually labeled landmarks less than 2.2 mm (DTI resolution), while in the AD patients, 70% of deformed landmarks had distance to the manually labeled landmarks less than 2.2 mm (Figure 7 in [21]). The test-retest reliability of the landmark placement was 1.58±0.60mm, suggesting that the segmentation quality approached the accuracy of this measurement.

**Table 1:** Demographic and clinical information.

	CON	AD
N	19	13
Age (SD)	76.5 (5.5)	73.5 (6.7)
MMSE	28.8	21.9
CDR-SB (SD)	0 (0)	5.77 (2.19)

Key: SD — standard deviation; CON — healthy controls; AD — Alzheimer's disease; MMSE — mini-mental state examination; CDR-SB — Clinical Dementia Rating-Sum of Boxes.

### 2.1 Volumes and Shapes of the White Matter Tracts

In traditional volumetric analysis, we examined group differences in the volume of each white matter tract between the healthy control subjects and the patients with AD using linear regression. After controlling the total intracranial volume, left cingulum in the hippocampus (CgH) showed significant white matter loss in AD (uncorrected p-value: p = 0.0111). But this did not hold up using Bonferroni correction for multiple comparisons at a significance level of 0.05 (p-value threshold = 0.05/23 = 0.0022). No group difference was found in the other white matter tracts. Using left CgH volume as feature, LDA leave-one-out cross validation yielded a classification accuracy rate of 65.6% (specificity: 78.9%; sensitivity: 46.2%) and F-score of 0.732.

**Figure 3:**
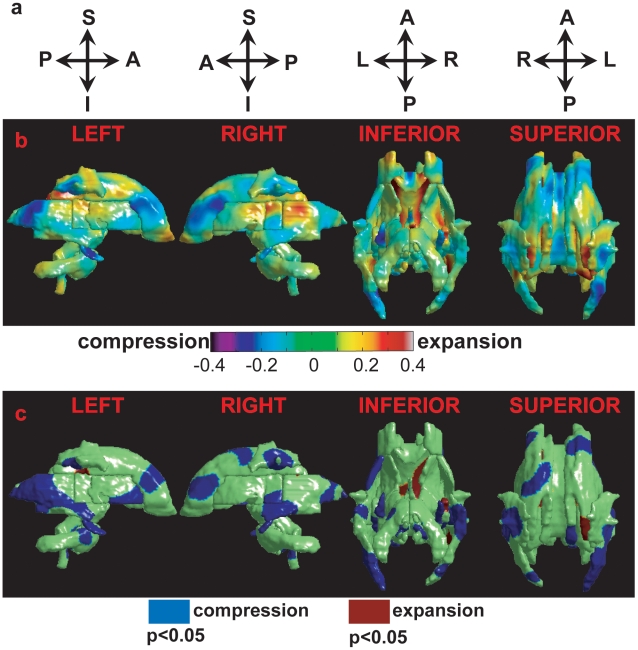
Deformation maps of the white matter tracts. Row (a) illustrates the anatomical orientation of the corresponding column. Row (b) shows the group difference map in the log-Jacobian determinant between the healthy control subjects and patients with AD. Warm color denotes regions with shape expansion in the AD group; while cool color represents regions with shape compression. Row (c) illustrates shape abnormalities of the deep white matter tracts in the patients with AD relative to the healthy control subjects. Blue denotes the regions with significant surface compression in the AD group compared with the control group, while red colors the regions with significant surface expansion in the AD group relative to the control group. Left, right, inferior, and superior views are respectively illustrated from the left to the right.

Figure 3(b) illustrates the average difference in the surface deformation maps between the groups of healthy controls and patients with AD. Regions with negative values are compressed in the AD group, while regions in positive values are expanded. After controlling for the total intracranial volume, linear regressions found pronounced regionally-specific shape abnormalities of the deep white matter tracts in patients with AD when compared with the healthy controls (Figure 3(c)). Permutation tests confirmed the overall significance of p = 0.0195. Compared with the healthy controls, the shape compression in the patients with AD occurs in bilateral sagittal stratum tract (SS), anterior corona radiata (CR), and cingulum in the hippocampus (CgH). The shape compression also occurs in left anterior external capsule (EC) and right superior longitudinal fasciculus (SLF). Moreover, the shape expansion in the patients with AD occurs in the posterior of the commissural tract (CC), which well corresponds to the expansion of the lateral ventricles in AD. When considering the deformation map as whole, only the 2^nd^ PC showed significant difference in the shapes of the white matter tracts between the control subjects and the patients with AD (p = 0.0002). Using it as feature, LDA leave-one-out cross validation yielded a classification accuracy rate of 81.3% (specificity: 89.5%; sensitivity: 69.2%) and F-score of 0.850.

### 2.2 Mean FA and FA map of the White Matter Tracts

In traditional ROI-based analysis, mean FA values were computed within the ROIs of individual white matter tracts. Compared with healthy control subjects, patients with AD showed reduction of mean FA values in bilateral fornix (uncorrected p-values, left: p = 0.0184; right: p = 0.0040), left corona radiata (uncorrected p-value, p = 0.0458), right limb of internal capsule (uncorrected p-value, p = 0.0030), right external capsule (uncorrected p-value, p = 0.0110). But these findings did not hold up using Bonferroni correction for multiple comparisons at a significance level of 0.05 (p-value threshold = 0.05/23 = 0.0022). No group difference in mean FA value was found in the rest of the white matter tracts. Using mean FA values in the tracts with significant group difference as features, LDA leave-one-out cross validation yielded a classification accuracy rate of 62.5% (specificity: 68.4%; sensitivity: 53.8%) and F-score of 0.684.

**Figure 4:**
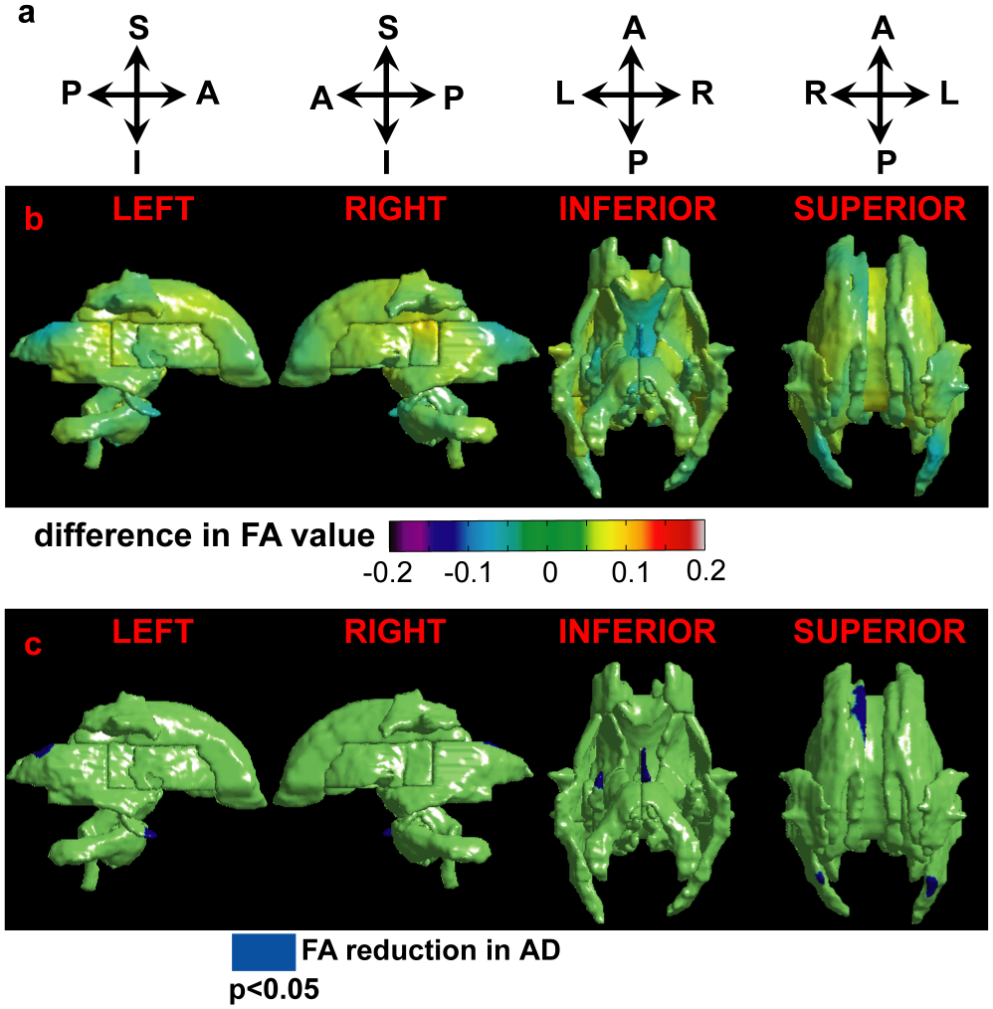
FA maps of the white matter tracts. Row (a) illustrates the anatomical orientation of the corresponding column. Row (b) shows the group difference in the FA map between the healthy control subjects and patients with AD. Warm color denotes regions with increased FA in the AD group; while cool color represents regions with reduced FA in the AD group. Row (c) illustrates FA abnormalities in the patients with AD relative to the healthy control subjects. Blue denotes the regions with significant FA reduction in the AD group compared with the control group. Left, right, inferior, and superior views are respectively illustrated from the left to the right.

Figure 4(b) illustrates the average difference in FA between the healthy control subjects and patients with AD. Regions in cool color are FA reduction in the AD group, while regions in warm color are FA increase in the AD group. Linear regressions found pronounced regionally-specific FA abnormalities of the deep white matter tracts in patients with AD when compared with the healthy controls (Figure 4(c)). Permutation tests confirmed the overall significance of p = 0.0375. Compared with the healthy controls, the FA reduction in the patients with AD occurs in bilateral SS, left anterior CR and CgH, right fornix. When considering the FA map as whole, only the 2^nd^ and 5^th^ PCs showed significant difference in the FA map of the white matter tracts between the control subjects and the patients with AD (2^nd^ PC: p = 0.0094; 5^th^ PC: p = 0.047). Using these two PCs as features, LDA leave-one-out cross validation yielded a classification accuracy rate of 71.9% (specificity: 78.9%; sensitivity: 61.5%) and F-score of 0.769.

### 2.3 Clinical Relationship

**Figure 5:**
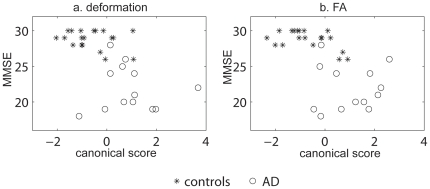
Clinical relations with the shape and FA of the white matter tracts. Panel (a) shows the relation between the MMSE and canonical score of the deformation map, while panel (b) illustrates the relation between the MMSE and canonical score of the FA map. Asterisks and circles respectively denote control and AD subjects.

Figure 5(a) illustrates the relationship of MMSE with the canonical scores of the deformation map. In the canonical analysis on the deformation map, patients with AD were associated with larger canonical scores, while healthy controls were associated with lower canonical scores. Pearson's correlation analysis revealed significant negative correlation between MMSE and the canonical score of the deformation map (r = −0.5680, p = 0.0007), suggesting that more severe shape abnormalities in the white matter tracts predicted lower MMSE scores.

Figure 5(b) illustrates the relationship of MMSE with the canonical scores of the FA map. In the canonical analysis on the FA map, patients with AD were associated with larger canonical scores, while healthy controls were associated with lower canonical scores. Pearson's correlation analysis revealed the significant negative correlation between MMSE and the canonical score of the deformation map (r = −0.6200, p = 0.0002), suggesting that more severe FA abnormalities in the white matter tracts predicted lower MMSE scores.

## Discussion

In this paper, the surface-based analysis was applied for assessing shapes and FA maps of the twenty three deep white matter tracts in patients with AD and healthy control subjects. The main contribution of this work was to construct the surface representation of deep white matter tracts. Using this surface model, patients with AD showed pronounced regionally-specific FA reduction and shape abnormalities mainly in the sagittal stratum and the cingulum (combination of corona radiate and cingulum in the hippocampus) when compared with healthy control subjects. The surface-based abnormal patterns of FA and shapes in the white matter tracts better distinguished patients with AD from healthy control subjects when compared with white matter volumes and mean FA values within each white matter tract (see Table 2). Furthermore, greater FA or deformation abnormalities of the white matter tracts were associated with a lower MMSE score.

**Table 2:** Classification results.

features	Volumes	Shape Pattern	Mean FA values	FA map
**accuracy rate**	65.6%	81.3%	62.5%	71.9%
**specificity**	78.9%	89.5%	68.4%	78.9%
**sensitivity**	46.2%	69.2%	53.8%	61.5%
**F-score**	0.732	0.850	0.684	0.769

The accuracy rate, specificity, sensitivity, and F-score of the classification are listed when the volumes, shape pattern, mean FA values, and FA map of the deep white matter tracts were respectively used as features in the classification.

Previous studies using structural MRI and PET revealed brain atrophy in the cingulum tract and showed its strong correlations with the hippocampal atrophy and hypometabolism of the mammillary bodies, thalamus, cingulate gyrus, parahippocampal gyrus, and hippocampus in AD. Using DTI, our study further confirmed the local volume loss and FA reduction of the cingulum tract in AD. Based on the white matter atlas, this cingulum tract contains fibers connecting the parahippocampal gyrus and hippocampus proper to the posterior cingulate cortex [20], [23], cortico-thalamic fibers, as well as cortico-cerebellar fibers [20]. This is in agreement with the finding that the hypometabolism in the limbic circuit results from the hippocampal formation atrophy via the cingulum tract disruption, which was suggested in previous studies [24]. It also interprets the striking discrepancy between the hypometabolic profile and the well described brain atrophy pattern in AD. Brain atrophy is characterized by the early involvement of the medial temporal lobe, subsequently spreading to the lateral temporal areas before extending to the cingulate and temporoparietal, frontal and occipital regions [25], consistent with the course of neurofibrillary degenerations [26]. Nevertheless, brain glucose metabolism alterations are characterized by the early involvement of the posterior cingulate cortex, subsequently spreading to the neighboring precuneus and temporoparietal regions [27], [28], [29].

Using DTI, our study for the first time reported the shape abnormality and FA reduction in the sagittal stratum tract that contains the inferior fronto-occipital fasciculus [30], the inferior longitudinal fasciculus (ILF), and the posterior thalamic radiation. DTI tractography showed that the ILF directly connects occipital branches related to areas V2 and V4 and anterior temporal branches related to the lateral temporal cortex, parahippocampal gyrus and amygdala [31]. The patient described by Ross [32] with a lesion apparently restricted to the ILF was unable to learn novel, non-verbalizable visual stimuli, despite the fact that visual information was able to reach the medial temporal lobe through other indirect pathways. One function of the direct pathway between the occipital and temporal lobes through the ILF is perhaps to prime medical temporal structures to facilitate the consolidation of visual memories. It therefore suggests that cognition impairment in visuospatial memory in AD could be due to disruption in the ILF. In addition to the ILF, the IFO, posterior thalamic radiation, EC, and SLF contain connections among the frontal, parietal, temporal, occipital lobes, and the cerebellum. Regional shape compression and FA reduction in these tracts may indicate the loss of neuronal axons or loss of connection with the cortex, suggesting a disruption in direct or indirect connectivity of the temporal lobe with the frontal, parietal, and occipital lobes as well as the cerebellum. As AD progresses, the propagation of the gray matter atrophy in AD from the temporal lobe to the frontal, parietal, and occipital lobes as well as the cerebellum may therefore be due to connectivity disruption in these tract regions.

Our findings support that AD is thought to reflect disrupted cortical connectivity. Integration of these shape abnormalities with white matter tissue properties (e.g. FA) provides new insights of abnormalities in white matter structures in AD. An open question is how these white matter changes in terms of geometry and tissue properties predict the disease when compared with the gray matter atrophy, cortical metabolism and functional connectivity, as well as underlying neuropathology that have been identified as image markers of AD [33], [34]. One possibility is that disruption in the white matter tracts near the temporal lobe represents the secondary consequence of the medial temporal lobe pathology. Subsequently, metabolic effects and brain atrophy represent the consequence of regionally-specific white matter tract disruption. Future research will be required to determine the relationship of white matter disruption with gray matter atrophy and cortical hypometabolism in AD, which may explain why certain regions of the brain show preferential vulnerability to AD as the disease progresses.

The data analysis in this study offers several strengths. The main contribution of this work is its construction of the surface representation of deep white matter tracts. The surface model is a natural representation for white matter tracts because the surface effectively summarizes the overall shape of the white matter tract whose variations relative to the atlas can be characterized as a scalar field of the surface through the surface diffeomorphic metric mapping [22], [35]. This provides a natural way for reducing the dimensionality of the shape deformation and offers an alternative to smoothing and performing statistical analysis based on geometric models of white matter tracts (e.g. [36], [37]). Furthermore, our surface-based analysis also offers a natural representation of diffusion properties as a function indexed over the surface manifold by projecting the diffusion measures (e.g. FA, diffusivity) onto it. Our clinical study with a small sample size demonstrates the feasibility and sensitivity of using the surface-based analysis to identify the regionally-specific shape and FA abnormalities in AD.

There are, however, several limitations to the proposed analysis framework. First, our atlas-based segmentation uses multiple image contrasts to drive the spatial normalization. It is possible to use the tensor information instead. Several methods have been postulated to use the full tensor for the spatial normalization of DTI [11], [12], [38]. They may provide additional benefits to further improve mapping accuracy. Second, the shape analysis introduced in this paper considers individual white matter tracts as whole, which cannot provide shape information (e.g. twist, split, interrupt) of individual white matter fibers. As the validity and reliability of the white matter fiber extraction is proven increasingly, we will accordingly be able to adapt our current analysis to investigating individual white matter fiber shape using the LDDMM curve mapping [39], [40]. Furthermore, the white matter bundles near the cortex are not included in this study. Our analysis framework could be applied to them when the atlas of these bundles is defined such as one introduced in [41].

In this study, the white matter atlas was built on a single-subject's DTI image. There is a reason for choosing this single-subject atlas rather than a population-averaged atlas. High-dimensional non-linear registration methods may not work properly with the population-averaged atlas in which the anatomical structures are blurred due to averaging. This is not a substantial issue for linear normalization, which is mostly driven by a large contrast change at the outside boundary of the brain, but the blurred internal structures could easily confuse high-dimensional non-linear registration [21]. Ideally, if the spatial normalization algorithm is perfect, the single-subject atlas simply serves as the origin of coordinates to measure anatomical variability and the location of the origin may not be important as long as we are interested in differences among groups. However, in reality, our atlas-based diffeomorphic segmentation assumes that the overall appearance of subjects' DTI images is sufficiently similar to that of this atlas so that the diffeomorphic mapping is feasible to deform the atlas to subjects. In studies where subjects with large tumor, or brain lesions are present, this atlas-based segmentation is likely not feasible. Recent advantages in DTI segmentation and higher resolution imaging may indeed make it possible to consistently label the tracts in individual subject images [42], [43], [44].

## Methods

### 4.1 Subjects

Two groups of subjects were included in the present study: (1) Healthy Controls (CON) (n = 19): subjects who were cognitively normal and had a Clinical Dementia Rating (CDR) of 0 [45], [46]; (2) Alzheimer's disease (AD) (n = 13): subjects who had mild AD, had a CDR = 1, and met NINCDS/ADRDA criteria for AD [47]. Participants were primarily recruited from two sources: the Clinical Core of the Johns Hopkins Alzheimer's Disease Research Center and memory clinics associated with Johns Hopkins Medicine Hospital. Subjects were excluded from enrollment if they were under age 55, had a history of a neurological disease other than AD, or a history of major psychiatric illness. Subjects were required to have a knowledgeable informant who could provide information about their daily function. Demographic and clinical information about the subjects is in Table 1.

#### Ethic Statement

All subjects provided written consent for participation in accordance under the oversight of the Johns Hopkins Institutional Review Board.

### 4.2 Data Acquisition and Preprocessing

High-resolution DTI data were acquired on a 3T Philips Achieva system using a single-shot EPI sequence with a SENSE parallel imaging scheme (Sensitivity Encoding, reduction factor = 2.5, TR = 6111.68 ms, TE = 71.0 ms). The imaging matrix was 96×96, with a field of view of 212×212 mm (nominal resolution of 2.2 mm), which was zero-filled to 256×256. Axial slices of 2.2 mm thickness were acquired parallel to the anterior–posterior commissure line. A total of 60 slices covered the entire brain and brainstem without gaps. The diffusion weighting was encoded along 30 independent orientations [48] and the b-value is 700 sec/mm^2^. Five additional images with minimal diffusion weighting (b = 33 sec/mm2) were also acquired (B0 images). Co-registered T_2_ weighted images were also acquired using a double spin echo sequence with a first echo time of 10.1ms, a second echo time of 96.0ms, and a repetition time of 3,000ms. The imaging matrix was 256×247, with a field of view of 240×210 mm. Axial slices of 3 mm thickness were acquired parallel to the anterior–posterior commissure line. A total of 48 slices covered the entire brain and brainstem without gaps.

To correct geometric distortion of the DTI due to B0-susceptibility differences over the brain, we followed the procedure detailed in [49]. The T_2_ weighted image was considered as anatomical reference. Within a subject, the deformation that carried its DTI to the T_2_ weighted image characterized the geometric distortion of the DTI. For this, intra-subject registration was first performed using Automated Image Registration (AIR) [50] to remove linear transformation (rotation and translation) between the 35 diffusion weighted images and T_2_ weighted image. Then, the LDDMM image mapping sought the optimal nonlinear transformation that deformed the B0 image to the T_2_ weighted image. Such diffeomorphic transformation was applied to every diffusion weighted image to correct the DTI nonlinear geometric distortion.

We aligned each subject's diffusion weighted images to the atlas anatomical space based on the affine transformation between the T_2_ weighted images of the subject and the atlas [50]. The diffusion tensor of the subject was determined by multivariate least-squares fitting. A fractional anisotropy (FA) map was computed based on the three eigenvalues of the tensor for quantifying the anisotropy of the deep white matter tracts.

### 4.3 Atlas-Based Diffeomorphic Segmentation of White Matter Tracts

For the atlas-based segmentation, we used the white matter atlas generated from the DT image of a single subject where the anatomical definition of each white matter tract followed the criteria described in [20]. The atlas consists of a collection of homogeneous volumes and smooth surfaces 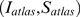 for individual white matter tracts, where 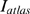 denotes the homogeneous volume and 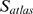 represents the surface at the boundary of 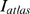. Figure 1 shows the deep white matter atlas in the volume (panels (a–c)) and surface (panels (d–g)) representations. The deep white matter tracts included in this atlas are listed in Text S1. The atlas is available online in an in-house program ROIeditor (www.mristudio.org).

Given the DT image of a subject, its white matter tracts are assumed to be generated based on the atlas via a flow of diffeomorphisms (one-to-one, reversible smooth transformations), solutions of ordinary differential equations 
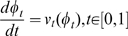
, where 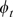 is a diffeomorphic flow. This flow starts from the identity map 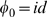, and is associated with velocity field 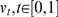. The topological and global shape properties of the atlas are transformed into the subject anatomical coordinates by solving the large deformation diffeomorphic metric mapping (LDDMM) algorithm [21] defined as
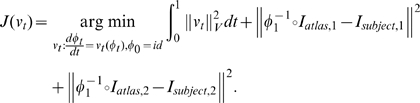
(1)The matching cost 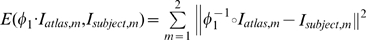 quantifies the intensity similarity between the deformed atlas and the subject, where 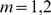 indexes image modality. In particular, we chose 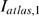 and 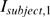 to respectively be the FA images of the atlas and the subject for controlling the image alignment in the white matter region, while 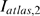 and 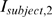 were respectively the images without diffusion weighting (b = 0) of the atlas and the subject for well matching the global shapes of the brain and the gray matter. The integrated norm 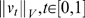 of the velocity field is the geodesic length of the curve that connects the atlas and the subject in the shape space. To ensure the curves are flows of diffeomorphisms, 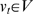 is a Hilbert space of smooth vector fields with norm square 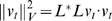 (see [51] for specific requirements). 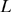 is a differential operator defined as 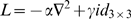, where 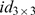 is a 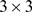 identical matrix and 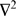 is the Laplacian operator. 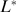 denotes as adjoint of 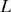. The 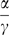 ratio affects the elasticity of the transformation. The matching quality improves as the ratio decreases [21]. In our study, we took a three-step cascading approach with a decreasing 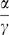 of 0.01, 0.005, and 0.0025 in the LDDMM mapping to gradually improve the matching quality. This procedure ensures that there is only a small amount of required transformation at each step up to 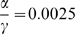. Denoting the surface representation of the atlas white matter tracts as 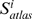, 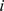 indexed the white matter tract (Figure 1). The deformed atlas segmentations are therefore given by 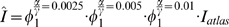; the shapes of the segmented white matter tracts are given by transforming the atlas surfaces under the same mapping 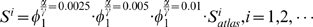.

### 4.4 Shape Analysis on the White Matter Tracts

To understand the shape variation of the white matter tract, 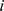, across subjects, we seek the optimal diffeomorphic transformation, 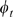, that connects 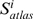 and 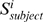. Such a transformation can be found through the LDDMM-surface mapping algorithm [22], [35] in the form of
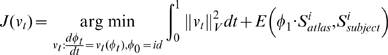
(2)where 
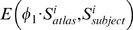
 quantifies the geometric similarity between the deformed atlas, 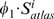, and the subject, 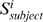 based on the closeness of normal vectors of the two surfaces. The mathematical form of 
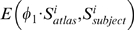
 was detailed in [22], [35]. To give this paper a sense of completion, we briefly introduced 
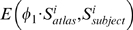
. The surface of the white matter tract embedded in 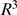 was assumed to be a two-dimensional manifold in the sense that the neighborhood of every point on the surface is equivalent to a two-dimensional plane in Euclidean space. Such a plane can be uniquely defined by a point and a vector originated at this point and normal to the plane. Therefore, we can represent a triangulated surface as 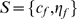, a set of points and normal vectors, where 
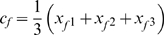
 is the center of triangle 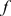 on 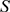 with three vertices 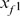,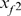,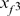 and 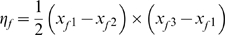 is the normal vector to 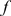 at location 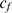. The symbol
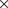
denotes cross product [22], [35].

Now we defined 
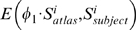
 for registering surfaces in the LDDMM setting based on their position and normal vectors. Let 
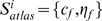
 and 
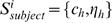
 be the atlas and subject triangulated surfaces represented by center points of triangles on the surface and their corresponding normal vectors. Denote the deformed atlas surface 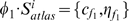, where 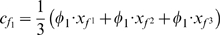 is the center of deformed triangle 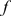 and 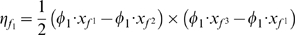 is the normal vector to deformed triangle 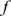 at location 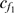. Let 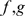 be indices of triangles on the surface 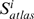 and 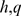 be indices of triangles on the surface 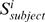. 
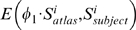
 is given in the form of
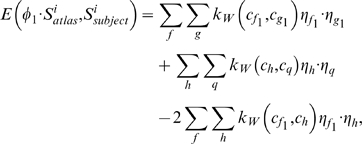
where 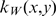 is a kernel and defined as an isotropic Gaussian kernel matrix, 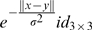. 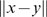 denotes Euclidean distance between points 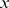 and 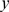 and 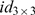 is a 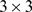 identical matrix. The first two terms are intrinsic energies of the two surfaces 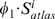 and 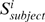. The last term gives penalty to mismatching between normal vectors of 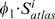 and 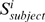.

The log-Jacobian determinant of the deformation was computed at every location of the atlas coordinates for each subject and was used to examine group differences (e.g., AD vs. controls) in shape. It is a smooth function over 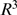 that indicates the ratio of the volume of subject's white matter tract to that of the atlas in a logarithmic scale. Positive values correspond to the surface expansion of a subject's white matter tract relative to the atlas, while negative values denote the surface compression of subject's white matter tract relative to the atlas. We shall term it as “surface deformation map” throughout the paper.

### 4.5 Surface-Based FA map of the White Matter Tracts

Obtaining a surface-based representation of FA requires a reduction of dimensionality, the assignment of the FA data in the 3D volumes of the white matter tracts to locations on their surfaces. We considered two steps that contribute to the mapping of a voxel in the volume of the white matter tract to a vertex on its corresponding surface. The first step was to find the proper association between the voxels in the white matter tract volume and its surface based on their Euclidean distance. Each vertex on the surface was thus associated with a set containing the voxels that have the shortest distance to this vertex. In the second step, FA value at this vertex was computed as averaged FA value over the set of its associated voxels.

### 4.6 Statistical Analysis

At each point on the tract atlas surface, the surface deformation map was modeled using linear regression with diagnosis as the main factor and the total intracranial volume as a covariate. The surface-based FA map was also modeled using linear regression with diagnosis as the main factor. The statistical results were corrected for multiple comparisons using permutation tests to determine the overall significance of the statistical maps. In each permutation trial, diagnostic labeling was randomly assigned to each subject and the number of points with significant main effects (p<0.05) was recorded. After 10,000 permutation trials, the overall significance was computed as the fraction of the time the suprathreshold area was greater in the randomized maps than the real effect [52].

Principal component analysis (PCA) [53] and linear discriminant analysis (LDA) [54] were applied for examining how well the FA maps and the deformation maps can distinguish the patients with AD from the healthy control subjects. PCA was first employed to reduce the dimensionality of the FA maps or the deformation maps. To identify the principal components (PCs) that significantly contributed to group differences, we first examined the two-sample *t*-test on each PC. A set containing PCs with corresponding p-value less than 0.05 was then chosen as feature space in LDA. Leave-one-out cross validation was used to examine the LDA performance. Classification accuracy rate, sensitivity, specificity, as well F-score were computed as quantitative evaluation of the LDA classification.

To examine the correlation of the FA map with the clinical measure of MMSE, PCs selected from the classification using the FA map were used to generate canonical scores. The canonical analysis was designed to score the control and AD subjects along the dimension that showed the difference between these two groups. More specifically, using a general linear model with the PC scores as dependent variables, and group as predictor variable, the canonical analysis computed the first eigenvector of matrix 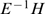, where 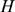 was the sum of squares and cross-products (SSCP) matrix associated with the contrast between the control and AD subjects, and 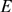 was the SSCP matrix of the model residuals (derived from the full model using all subjects). A canonical score was obtained for each subject by applying the weighting coefficients in the eigenvector to the original dependent variables (i.e., the PC scores). Pearson's correlation analysis was performed on the canonical scores with MMSE scores. We repeated this analysis for investigating the correlation of the deformation map with MMSE.

## Supporting Information

Text S1 Anatomical definition of white matter tracts.
(0.03 MB DOC)
