# Supplementary material for: Surface-Based Analysis on Shape and Fractional Anisotropy of White Matter Tracts in Alzheimer's Disease
Source: PLoS One. 2010 Mar 22;5(3):e9811. doi: 10.1371/journal.pone.0009811 (PMC2842443; doi:10.1371/journal.pone.0009811)
Supplement: Text S1 — Anatomical definition of white matter tracts. (0.03 MB DOC) [file pone.0009811.s001.doc]

**Anatomical Definition of White Matter Tracts**

The anatomical definition of the deep white matter tracts are detailed in (Mori et al. 2008). Figure 1 illustrates these white matter tracts in the volume and surface representations. For the completeness of the paper, we briefly describe what tracts are included in each surface. Note that we can define the following tracts in both hemispheres except the commissural tract.

1. Commissural tract (CC) contains the anterior commissure and the corpus callosum.
2. Corona Radiata (CR) contains the anterior, superior, posterior corona radiata, and cingulum at the axial level of the splenium of the corpus callosum.
3. External Capsule (EC) is located lateral to the internal capsule and contains association fibers.
4. Limb of Internal Capsule (LIC) contains the anterior and posterior limbs of internal capsule, as well as retrolenticular part of the internal capsule, which is a major tract of the thalamic radiation.
5. Sagittal Stratum (SS) contains the inferior fronto-occipital fasciculus, the inferior longitudinal fasciculus, and posterior thalamic radiation.
6. Superior Longitudinal Fasciculus: (SLF) contains connections between the frontal, parietal, occipital, and temporal lobes.
7. Fornix (FX) has the connection to the limbic system.
8. Stria Terminalis (ST) is related to the limbic system and connects to the amygdala.
9. Cingulum in the Hippocampus (CgH) is located at the axial level of the splenium of the corpus callosum.
10. Middle Cerebellar Peduncle (MCP) initiates from the pontine nuclei and carries information between the cortex and cerebellum.
11. Peduncle (PT) contains the inferior and superior cerebellar peduncle, the medial lemniscus, and cerebral peduncle. It carries information from the spinal cord and the medulla to the cerebellum and to the thalamus.
12. Corticospinal tract (CT) contains corticopontine and corticobulbar tracts.
